# Factors influencing adherence to addiction treatment in women with alcohol dependence and families at social risk

**DOI:** 10.1192/j.eurpsy.2026.10806

**Published:** 2026-07-31

**Authors:** E. Tiuliandina, T. Agibalova, A. Masyakin, E. Kharitonenkova

**Affiliations:** 1 Moscow Research and Practical Centre for Narcology of the Moscow Healthcare Department; 2 Peoples’ Friendship University of Russia named after Patrice Lumumba; 3 Federal State Budgetary Educational Institution of Higher Professional Education «Russian Medical Academy of Continuous Professional Education» of the Ministry of Health of the Russian Federation; 4 Central State Medical Academy of the Оffice of the President of the Russian Federation, Moscow, Russian Federation

## Abstract

**Introduction:**

Treatment outcomes in alcohol dependence are largely determined by patient adherence. Women from socially disadvantaged families frequently fail to recognize alcohol-related problems and neglect specialized care, which may result in parental rights restriction. This study examined clinical, psychological, and social predictors of adherence in this vulnerable group.

**Objectives:**

To identify factors influencing adherence to addiction treatment in women with alcohol dependence and family dysfunction.

**Methods:**

The sample included 71 women with alcohol dependence (F10.2), mean age 40 years (range 27–56), whose families were under child protection supervision due to maternal drinking (mean supervision duration 3.5 years, range 0–20). Indicators of adherence were regular clinic attendance, pharmacotherapy compliance, attitude toward medication (Drug Attitude Inventory, DAI-30), and current remission duration. Statistical analyses included Fisher’s exact test and Pearson’s correlation coefficient.

**Results:**

Prior hospitalization in addiction wards strongly predicted pharmacotherapy adherence (OR=5.0, p=0.003). Additional associations were found with education below secondary level (OR=3.0, p=0.051), inpatient rehabilitation (OR=2.5, p=0.086), multiparity (≥3 children) (OR=2.8, p=0.075), and child removal by authorities during treatment (OR=3.2, p=0.144). A positive medication attitude correlated with fewer addictive cognitions (McMullin Addiction Thought Scale; r=-0.5, p<0.001), readiness for change (SOCRATES; r=0.4, p<0.001) - particularly problem recognition (r=0.5, p<0.001) and action taking (r=0.3, p=0.005), and illness perception as life-threatening (Brief IPQ; r=0.3, p=0.004). Personality traits showed minor effects: borderline (r=0.2, p=0.167) and absence of antisocial traits (r=-0.2, p=0.139). Longer remission was linked to stable employment (ASI; r=-0.3, p=0.004), older age (r=0.2, p=0.089), longer drinking history (r=0.2, p=0.085), and maximum past remission (r=0.3, p=0.010). Outpatient rehabilitation (OR=7.1, p<0.001) and participation in peer-support groups (OR=9.0, p=0.019) were associated with regular clinic attendance.

**Conclusions:**

Among women with alcohol dependence and family dysfunction, adherence to addiction treatment is primarily determined by critical awareness of the disorder and motivation for change, rather than by external threats such as potential parental rights deprivation.

**Disclosure of Interest:**

None Declared

